# New Hampshire Veterinary Medical Association

**Published:** 1896-06

**Authors:** L. Pope

**Affiliations:** Secretary


Secretary.

### NEW HAMPSHIRE VETERINARY MEDICAL ASSOCIATION.

THE annual meeting convened in the Eagle Hotel, Concord, Tuesday, May 5th, at 11 A.M., with Vice-President Dr. Hart in the chair.

Drs. Hart, Maguire, Lilico, Abbott, Wilkinson, and Pope answered to roll-call. The following officers were elected by ballot for the ensuing year: President, T. G. Lilico, M.R.C.V.S.; Vice-President, F. C. Wilkinson, D.V.S.; Secretary and Treasurer, L. Pope, Jr., M.D.V.

Following the election of officers, discussion ensued over the action taken by Dr. Geo. Bailey, of the Maine Cattle Commission, against New Hampshire veterinarians.

The Secretary was instructed to notify the Massachusetts Cattle Commission of non-qualified men making tests with tuberculin now acceptable to that Board.

A paper was read by Dr. Abbott on "Homœopathy in Veterinary Medicine," which was most excellent in its preparation and delivery. Discussion following showed a strong feeling against the new school.

The meeting adjourned at 1 P.M. until September meeting.

The reports of Secretary and Treasurer were read and accepted.

L. POPE, JR., M.D.V.,
Secretary.

